# A systematic review of the effectiveness of psychological interventions in organ transplantation

**DOI:** 10.3389/fpsyg.2025.1716455

**Published:** 2025-12-19

**Authors:** Concetta De Pasquale, Maria Luisa Pistorio, Alberto Sardella, Massimiliano Veroux, Vittorio Lenzo, Micol Di Bella, Flavia Coco, Giuseppa Maria Stella Catania, Antonino Grasso, Alessia Giaquinta, Maria Catena Ausilia Quattropani, Pierfrancesco Veroux

**Affiliations:** 1 Vascular Surgery and Organ Transplant Unit, Department of General Surgery and Medical-Surgical Specialties, University Hospital of Catania, Catania, Italy; 2 Department of Educational Sciences, University of Catania, Catania, Italy; 3 Organ Transplant Unit, Department of Surgical and Medical Sciences and Advanced Technologies, University Hospital of Catania, Catania, Italy; 4 Unit of Pediatric Clinic, Department of Clinical and Experimental Medicine, University of Catania, Catania, Italy

**Keywords:** clinical psychology, mental health, organ transplantation, psychological intervention, systematic review

## Abstract

**Background:**

Organ transplantation profoundly affects not only patients’ physical health but also their psychological, emotional, and social well-being. Psychological interventions may support treatment adherence, emotional adjustment, and quality of life, yet the existing literature remains fragmented and methodologically heterogeneous.

**Objectives:**

This systematic review aimed to examine the available evidence on the effectiveness of psychological interventions in solid organ transplant recipients, with particular attention to outcomes related to anxiety, depression, stress, adherence, and quality of life.

**Methods:**

Following the PRISMA 2020 guidelines and with the protocol registered on PROSPERO (ID 1165280), a comprehensive search was conducted across PubMed, Scopus, and Web of Science. Inclusion criteria targeted studies involving adult solid organ transplant recipients receiving validated psychological or psychosocial interventions. Twenty-four studies met the eligibility criteria. Data extraction included demographic characteristics, organ type, psychological outcomes, intervention type, and duration. Relevant data, including quantitative findings and effect sizes where available, were extracted and synthesized.

**Results:**

The included studies revealed a heterogeneous yet coherent body of evidence supporting the relevance of psychological interventions in transplant care. Various approaches—including cognitive-behavioral, psychoeducational, mindfulness-based, expressive, and psychodynamic interventions—were associated, to different extents, with improvements in emotional well-being, adherence, and quality of life. The diversity of designs and outcomes, however, limits direct comparisons and precludes firm conclusions about relative effectiveness.

**Conclusion:**

Psychological interventions represent a key component of comprehensive transplant care, contributing to patients’ emotional adjustment, adherence, and overall quality of life. Nonetheless, current evidence is constrained by small sample sizes, methodological heterogeneity, and short follow-ups. Future research should prioritize multicenter and longitudinal studies using standardized psychological and clinical outcomes, in order to strengthen the current evidence base and support the systematic integration of psychological interventions into transplant care.

**Systematic review registration:**

Identifier PROSPERO (ID1165280).

## Introduction

Organ transplantation represents one of the greatest achievements of contemporary medicine, radically changing the prognosis of many chronic and terminal illnesses and restoring to patients both life expectancy and quality of life (Annema, 2023). The procedure, which consists of replacing a failing organ with a healthy one from a deceased or living donor, often constitutes the only chance of survival for these patients. Beyond its extraordinary surgical complexity and the immunological challenges associated with rejection, transplantation is best understood as an existential event, carrying profound psychological, emotional, and social implications (Abbey and Farrow, 1998a,b; Grigis, 2018a,b; Telles-Correia and Coelho, 2025).

The transplant experience should not be reduced to a surgical act but rather conceived as a complex and ongoing process that begins in the preoperative phase—often marked by waiting-list enrollment—and continues into the postoperative period, when patients must adapt to their new condition of life. Throughout this trajectory, patients face a series of demanding psychological experiences: anxiety linked to uncertainty and the possibility of not receiving a compatible organ in time, fear of surgery and its potential complications, difficulties in integrating the transplanted organ into their body image, and ambivalent feelings toward the donor (Abbey and Farrow, 1998a,b; Consoli, 2004a,b; Schulz and Kroencke, 2015). Gratitude and hope often coexist with guilt and distress, especially when the graft derives from a living donor, creating a sense of indebtedness that can be difficult to elaborate (Baines et al., 2002a,b; Zhao et al., 2021). For living organ donors themselves, recent evidence indicates significant rates of depression and anxiety, underscoring that the transplant process impacts donors as well (Ong et al., 2021; Brito et al., 2019; Asanova et al., 2025).

The postoperative phase, far from being solely a period of relief, introduces additional psychological challenges. Patients must adapt to a “modified” body, integrate an “alien” organ into their sense of self, and cope with lifelong immunosuppressive therapies that can cause significant side effects—such as weight gain, tremors, or aesthetic changes—while contributing to anxiety, depression, and difficulties with body-image acceptance (Grigis, 2018a,b). In some cases, a phenomenon of “psychological rejection” occurs, reflecting unconscious resistance to incorporating the graft into one’s identity (Consoli, 2004a,b; Látos et al., 2016). Indeed, research indicates that mood disturbances and reduced quality of life can persist even after successful transplantation (Basiri et al., 2020; Asanova et al., 2025).

Against this background, psychological support plays a crucial role across the entire transplant trajectory. A growing body of evidence indicates that emotional and psychological states directly affect treatment adherence, stress management, and, indirectly, clinical outcomes (Baines et al., 2002a,b; Gross et al., 2010). Patients struggling with depressive symptoms or a lack of acceptance are at higher risk of nonadherence to immunosuppressive regimens, which may result in rejection or serious complications (Weinrieb and Lucey, 2007). Conversely, targeted psychological interventions can promote acceptance, foster resilience, and improve long-term quality of life (De Pasquale et al., 2020).

A variety of psychological and psychosocial interventions have been explored in the literature. Over the years, several forms of psychological intervention have been proposed – ranging from individual and group psychotherapy (Consoli, 2004a,b; Caviglia, 2013), to cognitive-behavioral techniques (Epstein et al., 2019a,b; Parker Gregg, 2021), psychoeducation (Hodges et al., 1995), and approaches based on mindfulness or expressive therapies (Gross et al., 2010; Heather and Stockwell, 2004). However, the available studies remain limited and are often methodologically heterogeneous.

A recent meta-analysis of psychosocial interventions in transplant populations confirmed some beneficial effects but also highlighted the considerable heterogeneity and limited methodological quality of existing studies (Sambucini et al., 2022). Specifically, the authors reported that while psychological interventions such as cognitive-behavioral therapy, psychoeducation, and mindfulness-based programs are associated with improvements in emotional well-being and adherence, most available studies are limited by small sample sizes, lack of randomization, and short-term follow-up. These findings underscore the need for more rigorous and longitudinal research designs capable of assessing the long-term impact of psychosocial support on both psychological and clinical outcomes.

Building on these gaps, the present systematic review not only aims to synthesize and critically evaluate the available evidence but also to propose a future research agenda that addresses current methodological limitations. Specifically, this review seeks to:

1. Identify and categorize the main types of psychological and psychosocial interventions described in the literature;
2. Evaluate their effectiveness in reducing anxiety, depression, and stress, as well as improving treatment adherence and quality of life;
3. To compare different therapeutic approaches—including cognitive-behavioral therapy (CBT), psychoeducation, mindfulness-based interventions, and expressive therapies—in order to identify which strategies appear to be the most effective;

To assess the impact of psychological interventions on quality of life and social wellbeing, as these dimensions are essential for the full reintegration of transplant recipients into everyday life.

## Objective

The primary objective of the present systematic review is to provide an in-depth examination of the existing evidence on the effectiveness of psychological interventions in organ transplant recipients, with a particular focus on the psychological, emotional, and social dimensions that influence their post-operative recovery process.

### Request objective

This review aims to examine the psychological interventions applied in the context of organ transplantation, with the following specific objectives:

1. To clarify the extent to which these interventions can alleviate psychological symptoms such as anxiety, depression, and stress, and promote a more adaptive adjustment to the transplantation experience;
2. To evaluate the role of psychological interventions in enhancing adherence to pharmacological treatment, a critical determinant of clinical prognosis in transplant patients;
3. To compare different therapeutic approaches — including cognitive-behavioral therapy (CBT), psychoeducation, mindfulness-based interventions, and expressive therapies — in order to identify which strategies appear to be the most effective;
4. To assess the impact of psychological interventions on quality of life and social wellbeing, as these dimensions are essential for the full reintegration of transplant recipients into everyday life.

## Methods

**Figure 1:**
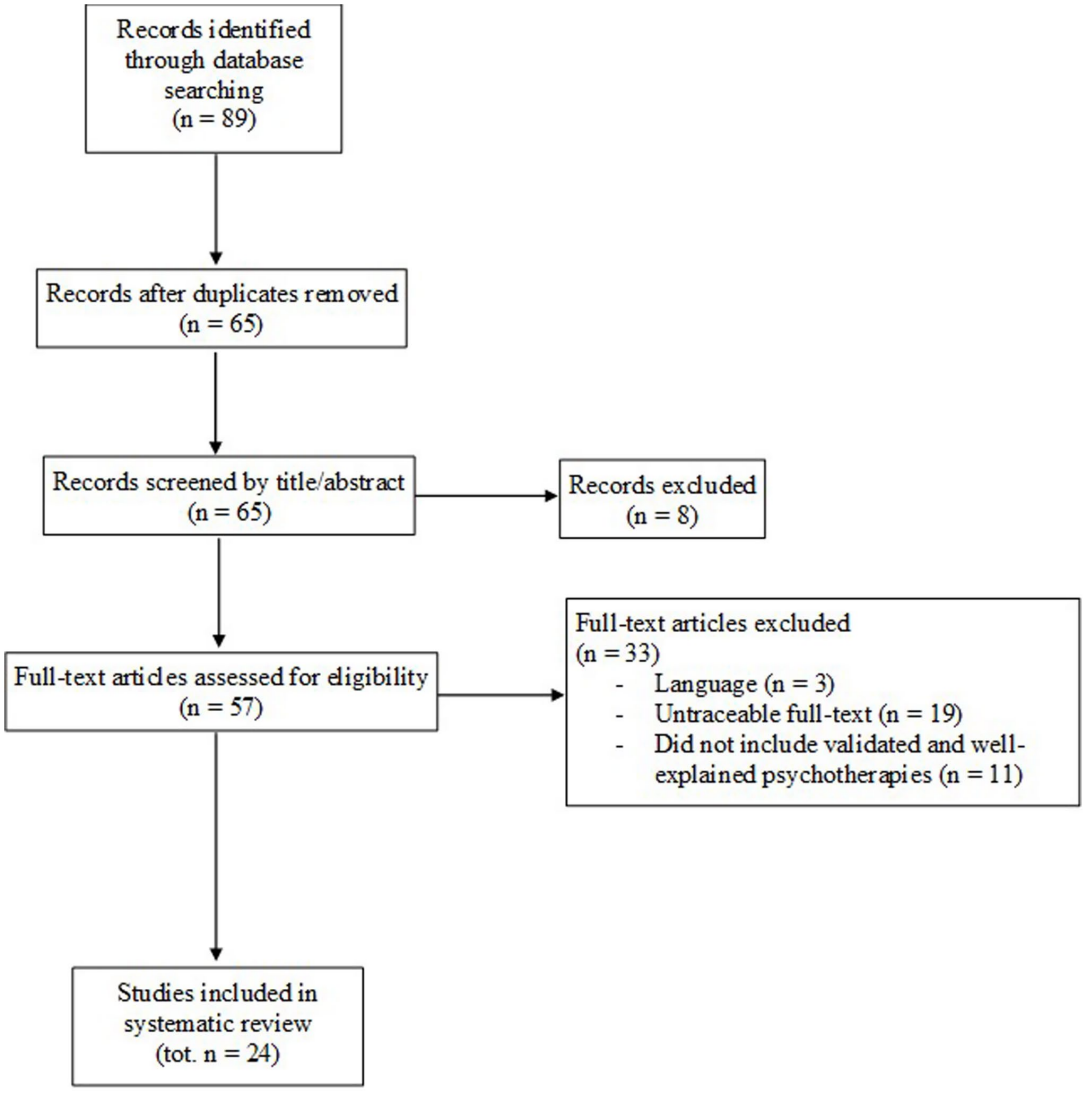
PRISMA flow chart.

This systematic review was conducted in accordance with the Preferred Reporting Items for Systematic Reviews and Meta-Analyses (PRISMA 2020) guidelines to ensure completeness and transparency (Page et al., 2021), and the protocol was registered on PROSPERO with the ID 1165280. The PRISMA flow chart is also provided as Figure 1.

### Search strategy and data sources

The literature search was conducted in two main phases. Initially, the electronic databases PubMed, Scopus, and Web of Science were consulted using the following keywords in Boolean: [(psychotherapy OR “psychosocial treatment*” OR “psychological treatment*”) AND (“organ transplantation” OR transplantation)].

The aim was to identify all relevant studies addressing psychological and psychosocial interventions in solid organ transplant recipients.

### Inclusion and exclusion criteria

This systematic review applied predefined inclusion and exclusion criteria to ensure the selection of relevant and methodologically robust studies. The rigorous application of these criteria reduced the risk of bias and enhanced the reliability of the evidence considered. Age of the population. Only studies involving adult participants (≥18 years) were included. This decision was based on the recognition that adult transplant recipients present distinct psychological and social needs compared to pediatric patients, who require specific interventions.

Studies including participants under the age of 18 were excluded. Type of transplant. The review focused exclusively on studies involving solid organ transplants (heart, liver, kidney, lung). Studies concerning stem cell, tissue, or other non-solid transplants were excluded, as these populations are characterized by different psychological dynamics. Type of intervention. Only studies reporting validated and clearly described psychological or psychosocial interventions were included. Studies lacking sufficient detail on the intervention or employing non-validated approaches were excluded.

Only studies published in English or Italian were included in this review. Articles written in other languages were excluded due to the lack of available translations and to ensure consistency in data extraction and interpretation. Moreover, several potentially relevant studies were excluded because the full texts were not accessible. In these cases, the authors were contacted to request the full manuscripts, but only a limited number responded, making it impossible to include those studies in the final analysis.

The systematic application of these criteria ensured the inclusion of studies of higher quality and relevance to the objectives of the review.

### Eligibility screening

The eligibility of the studies was assessed through a three-step screening process independently conducted by two authors (VL, AS). The evaluation was carried out sequentially on titles, abstracts, and finally full texts. Any disagreements regarding inclusion were discussed and resolved with the involvement of a senior author (MV). Out of 89 articles initially identified, 24 met the inclusion criteria and were included in the review. This dual independent screening procedure, combined with consensus resolution, ensured the reliability and consistency of the selection process.

### Data extraction

Data were extracted according to a coding protocol preliminarily defined and shared by all the authors. The extracted information included, wherever possible, the demographic characteristics of participants, the type of organ transplanted, the psychological outcomes assessed, the type of psychological intervention performed, and the duration of the intervention. All quantitative and descriptive findings are summarized in Table 1.

**Table 1:** Characteristics of included studies.

Authors	Population	Study design	Intervention	Follow Up	Measurement	Results
Abbey and Farrow (1998a,b)	Transplant candidates and support people (*n* = 16) Transplant patients and support people (*n* = 40) Adult solid organ transplant patients (kidney, liver, heart, lung)	Cohort study	Pre/post-transplant group therapy: psychoeducation (4 sessions, 10–16 pts), support (8 sessions), stress mgmt. (8), supportive-expressive (weekly, up to 24), CBT-based “Self-Management” (12 sessions). Led by psychosocial staff. Intake required only for expressive groups.	Not reported	SF-36 Illness Intrusiveness Rating Scale Mental Health Inventory Affect Balance Scale Life Satisfaction Rating	Reduced depression/anxiety, better QoL and emotional/social functioning (SF-36, MHI, ABS), less illness intrusiveness, and more positive affect. High satisfaction reported; early data support group CBT.
Baines et al. (2002a,b)	Adult kidney transplant patients (*n* = 49)	Cohort study	Individual psychotherapy: 12 weekly sessions, initiated within 3 months post-transplant	3–12-month follow-up	Beck Depression Inventory (BDI), self-report, assessed at baseline, post-treatment, and at 3-, 6-, 9-, and 12-month.	BDI scores dropped (26.6 → 18.9, *p* = 0.001), sustained at 12 months. Improved mood, functioning, and identity integration post-transplant.
Baines et al. (2004)	Kidney transplant patients (*n* = 89). Age of participants between 36 and 39 years old	Cohort study	12-week Systemic Integrative Psychotherapy (individual or group), starting within 3 months post-cadaver renal transplant. Same therapist and model for both arms.	12 months (3, 6, 9, 12)	Beck Depression Inventory (BDI), self-report. Administered pre-treatment, post-treatment, and at 3, 6, 9, and 12 months.	BDI scores improved more with individual therapy (26.6 → 18.9, *p* = 0.001) than group therapy (30.2 → 26.0, *p* = 0.01). Control group worsened over time.
Buchanan (1975)	Kidney transplant patients (*n* = 8) + support people age of participants from 20 to 55 years old	Cohort study	Weekly 1-h group sessions with transplant patients and family; focus on coping, education, and emotional support.	Not reported	Outcomes were assessed through participants’ subjective feedback at the end of the 10-session group intervention. No standardized psychometric instruments were used.	Improved emotional support, reduced isolation, and better post-transplant adjustment. Promoted peer learning and coping; positive subjective feedback despite no standardized measures.
Carrique et al. (2021)	Adult liver transplant patients (*n* = 44)	Cohort study	Relapse Prevention Therapy (RPT) which included 6–10 individual sessions with a therapist	Approximately 339 days (11 months)	Multidisciplinary psychosocial and medical assessments	Only 6.8% relapsed post-transplant—lower than unsupported patients. Psychological support improved adherence, reduced complications, and enhanced QoL and emotional stability.
Chisholm-Burns et al. (2013)	Kidney transplant patients (*n* = 150) average age of 52.06 years	Cohort study	Behavioral Adherence Contracts (BACs) over 1 year; pharmacist-led sessions every 3 months (baseline, 3, 6, 9, 12 months) focused on goals, barriers, support, reminders, and consequences of nonadherence.	Participants were followed for 1 year, with refill records collected for 3 months post-intervention to assess adherence	MEMS caps used to objectively monitor adherence to immunosuppressive therapy (IST).	Improved IST adherence; fewer hospitalizations (76.1% vs. 42.7%); reduced complications and healthcare costs.
De Geest et al. (2006)	Kidney transplant patients (*n* = 18) average age of 45,6 years	Cohort study	Home visit + 3 monthly follow-up calls focused on self-efficacy, EM feedback, problem-solving, medication routines, and social support.	3 monthly calls reviewing EM data	Electronic Medication Event Monitoring System (MEMS)	Improved adherence and awareness of immunosuppressive therapy in the intervention group (*p* = 0.6)
Epstein et al. (2019a,b)	Adult heart transplant patients (*n* = 12)	Cross sectional study	CBT via phone (4 sessions over −10 weeks); adapted Safren protocol; focused on coping, breathing, cognitive restructuring, activity planning.	4 biweekly sessions, final with symptom review and feedback.	Pre/post: HADS, PHQ-9, ITAS.	CBT uptake higher in symptomatic patients (5.67×, p = 0.006); 100% declined in-person, 70% preferred phone; protocol shortened and remote.
Freyberger et al. (1979)	Adult kidney transplant patients and living donors.	Cross sectional study	Psychiatric support in renal transplant unit via: Group therapy (every 3 weeks), indirect & direct psychotherapy (individual and with staff), crisis intervention and family therapy, balint-style team supervision.	Ongoing throughout hospitalization and outpatient care, no fixed duration	Self-report questionnaires: HADS (Hospital Anxiety and Depression Scale), SF-36 (QoL), STAXI-2 (anger expression), before/after.	Improved adaptation, coping with denial, psychosomatic symptom relief; interventions had stabilizing effects on self-esteem, frustration tolerance, and emotional processing. Outcomes supported psychiatric liaison model in transplant care.
Ghetti (2011)	Adult liver and kidney transplant patients (*n* = 29)	Cohort study	Weekly group music therapy (MT) sessions (90 min, 12 weeks) for kidney transplant recipients. Sessions included listening, active music-making, emotional expression, and sharing. Conducted by a trained music therapist in a supportive environment.	Post-intervention assessment immediately after the 12-week program. No long-term follow-up mentioned.	BDI (Beck Depression Inventory) for depression, STAI for anxiety, QoL scales for well-being. Assessments before and after intervention.	Significant reductions in anxiety (p = 0.005) and anger expression (*p* = 0.047); improvement in physical role functioning (*p* = 0.036). Intervention supported coping, reduced denial, enhanced emotional support, and alleviated psychosomatic symptoms.
Gross et al. (2009)	Solid organ transplant patients (*n* = 150) patients age 18 >	Cohort study	8-week Mindfulness-Based Stress Reduction (MBSR) program (weekly 2.5-h group sessions + 1 all-day retreat); compared with peer-led Health Education (HE) and Waitlist Control. MBSR included meditation, yoga, and group discussion tailored for transplant recipients.	8 weeks (end of intervention), 6 months, and 12 months.	Self-reported depression, anxiety, insomnia, and health-related quality of life; tracked via mailed surveys and participant diaries.	MBSR significantly reduced depression (*p* < 0.001), anxiety (*p* < 0.001), and insomnia (p = 0.001) compared to controls; improvements maintained at 6 and 12 months.
Heilmann et al. (2011)	Adult heart transplant patients (*n* = 51)	Cross sectional study	Supportive psychotherapy (SPT) offered to heart transplant (HTX) and ventricular assist device (VAD) patients (destination therapy, bridge-to-transplant, post-transplant), including individual, family, and liaison interventions by a psychologist during pre- and in-hospital post-op periods. Therapy includes crisis intervention, relaxation, and family support.	Contacts and psychological time tracked throughout hospitalization (pre−/post-op); HTXvad patients followed also between VAD implant and HTX. No post-discharge follow-up data.	Self-report questionnaire post-discharge assessing perceived helpfulness and usage of imagery; qualitative and quantitative items.	Adjustment disorders were most frequent (≈66%), followed by mild/moderate depression (≈15%). HTXvad patients showed higher emotional distress and therapy needs, requiring significantly more support than HTXprim (*p* = 0.046 for number of contacts; *p* = 0.006 for time spent). No significant difference in family therapy time (*p* = 0.253). About half of families used support therapy.
Hodges et al. (1995)	Lung transplant candidates (*n* = 16) Lung transplant recipients (*n* = 11) Family support members (*n* = 9) All adults	Cross sectional study	Bibliotherapy intervention using the book Surviving Transplantation, a psychoeducational self-help manual designed for lung transplant candidates, recipients, and their support persons. Participants received the book for a 2-week period, with no obligation to read it entirely. No therapist guidance was provided.	Follow up over a 12-month period. Therapy duration varied; s	Coping Strategies Inventory (CSI) Self-Directed Learning Readiness Scale (SDLRS) State–Trait Anxiety Inventory (STAI) Life Orientation Test (LOT) General Health Questionnaire (GHQ)	89% read the entire book; all participants found it helpful, with no negative emotional effects reported. Significant reduction in two coping domains: Emotional expression (*p* < 0.01) Social withdrawal (*p* < 0.0001) No change in levels of psychological distress (BDI, GHQ, STAI remained stable). Avoidant coping style (disengagement) predicted non-completion (p < 0.01). Older participants and those with higher support networks engaged more with the book (*p* < 0.05). Book rated positively (interesting, worthwhile, educational, easy) by the majority.
Hogan and Silverman (2015)	Solid organ transplant patients (*n* = 25) Age 18 >	Cohort study	Single 30-min music therapy session using Coping-Infused Dialogue with Patient-Preferred Live Music (CID-PPLM), alternating between live music and structured dialogues. Dialogues targeted surface conversation, local/global stressors, coping strategies, and generalization of skills, with live music performed on acoustic guitar based on patient choice.	Single-session design; outcomes measured immediately after session.	Global Mood Scale (adapted for real-time mood): measured positive and negative affect Visual Analog Scale for current pain (0–10)	No significant pretest differences were found. At post-test, the experimental group showed significantly better outcomes in positive affect (η^2^ = 0.198), negative affect (η^2^ = 0.422), and pain (η^2^ = 0.303), with medium-to-large effect sizes. These findings suggest that receptive music therapy may effectively improve mood and reduce pain in transplant recipients, especially when patient engagement is high.
Kober et al. (1990)	Adult liver transplant patients (*n* = 67)	Cohort study	Multidisciplinary psychological support provided before, during, and after liver transplantation; included individual consultations, group therapy, family involvement, and stress management. Focus on improving emotional adjustment, coping with illness, and patient education.	Regular psychological monitoring throughout transplant stages, including ICU, ward stay, rehabilitation, and up to 3 years post-transplant.	Used modified EORTC QoL questionnaire, STAI-X (anxiety), and structured interviews to assess general symptoms, emotional distress, and QoL across multiple timepoints (pre-op, 3, 6, 12, 24 months post-op).	A significant reduction in anxiety over time was observed in the intervention group (p = 0.046), particularly in the State–Trait Anxiety Inventory (STAI) scores. There was also a significant improvement in sleep quality in the intervention group over time (*p* = 0.016), measured using the Pittsburgh Sleep Quality Index (PSQI). No significant changes were observed in depressive symptoms between groups.
Lisson et al. (2005)	Adult liver transplant patients	Cohort study	A brief personalized psychological intervention delivered bedside before discharge to liver transplant recipients. Conducted by a psychologist (90–120 min session), it focused on enhancing intrinsic motivation, self-control, and self-efficacy; overcoming adherence barriers; reinforcing autonomy and commitment to care; and establishing a behavioral contract signed by patient, caregiver, and psychologist. Booster sessions provided as needed.	Adherence assessed at 1, 3, 6, and 12 months post-transplant,	Adherence evaluated through structured interviews, validated questionnaires, clinical coordinator reports, blood test results, and health service use records.	The intervention was positively received by patients, caregivers, and staff. Though no *p*-values were reported, qualitative data indicated improved awareness, motivation, and adherence behaviors during follow-up.
Madson and Silverman (2010)	Adult solid organ transplant patients (*n* = 51)	Cross sectional study	Single session of live music therapy (15–35 min) delivered in a hospital setting by a certified music therapist using guitar and voice. Sessions included patient-preferred music and interaction, and were tailored to post-transplant needs.	No long-term follow-up was conducted. Data were collected only pre- and post-session.	Pre-post design with self-reported 10-point Likert scales assessing relaxation, anxiety, pain, and nausea; observer-rated affect and verbalizations were also recorded.	Significant improvements in relaxation and anxiety (*p* < 0.001), pain (*p* < 0.01), and nausea (*p* < 0.05) after the session. Observer ratings showed increased positive affect and verbalizations (*p* < 0.001). All participants requested future sessions.
Stonnington et al. (2016)	Heart, liver, kidney/pancreas or stem cell transplant patients (*n* = 31) Caregivers (*n* = 18) All adults	Cohort study	The study tested a 6-week Mindfulness-Based Resilience Training (MBRT) program designed for both transplant patients (solid organ and stem cell) and their caregivers. T Sessions combined short mindfulness practices, psychoeducation, yoga/mindful movement, and experiential exercises. Each session included: A psychoeducational component, experiential mindfulness practices, mindful movement (e.g., yoga) Three groups were conducted over a year at the Mayo Clinic, each with 15–20 participants.	Outcomes were measured at baseline, 6 weeks, and 3 months post-intervention. There was no long-term follow-up beyond 3 months.	Perceived Stress Scale (PSS-10) Connor-Davidson Resilience Scale (CD-RISC) Patient Health Questionnaire (PHQ-9)—depression Generalized Anxiety Disorder-7 (GAD-7)—anxiety SF-36 – health-related quality of life Positive and Negative Affect Schedule (PANAS) Mindful Attention Awareness Scale (MAAS) Subjective sleep quality	Patients showed significant improvements from baseline to both 6 weeks and 3 months in: Perceived stress (*p* = 0.0011) Depression (PHQ-9) (*p* = 0.0025) Anxiety (GAD-7) (*p* = 0.0002) Negative affect (PANAS) (*p* = 0.0027) Positive affect (PANAS) (*p* = 0.0056) SF-36 Mental Component Summary (MCS) (*p* = 0.0247) Changes in resilience (*p* = 0.0852), mindfulness (*p* = 0.0593), sleep (*p* = 0.1748), and physical health (SF-36 PCS, *p* = 0.1317) were not statistically significant. Caregivers showed trends toward improvement in stress, anxiety, and mindfulness, but none reached statistical significance after correction for multiple comparisons. All participants reported improved well-being and most would recommend the program. No participant objected to the inclusion of mixed transplant types or both patients and caregivers in the same group.
Wagner-Skacel et al. (2023)	Kidney transplant patients (*n* = 22) Liver transplant patients (*n* = 19) Average age of 58,5 years	Cohort study	Multilevel step-guided psychotherapeutic program combining psychoeducation, individual sessions, group therapy, and daily short interventions (5–10 min) during hospital stay. Program tailored to individual psychological needs, addressing medication adherence, coping strategies, and emotional functioning.	6 months post-transplant follow-up.	Primary: Medication adherence measured monthly using the validated BAASIS questionnaire. Secondary: Clinical outcomes (infections, rejection, graft function), tacrolimus trough levels (CV%), and psychological assessments using OPD-SQS and ECR-RS at baseline and months 1–6.	Non-adherence significantly lower in intervention group (30%) vs. control (65.6%) at 6 months (*p* = 0.019). Intervention group had fewer hospital readmissions (*p* = 0.049) and lower TAC variability (*p* = 0.028), indicating better medication consistency.

### Quality assessment

The methodological quality of the included studies was evaluated using the Newcastle–Ottawa Scale (NOS).^1^ Given that most studies in our review adopted a cross-sectional design, we employed the adapted NOS version for cross-sectional studies, as recommended by Herzog et al. (2013). The standard validated NOS was applied to the remaining studies designed as case–control investigations.

Two authors (VL, AS) independently performed the quality assessment to identify potential sources of bias. Any disagreements were resolved through discussion with a senior author (MV). An overview of the quality assessment outcomes for all included studies is presented in Tables 2, 3.

**Table 2:** Quality assessment of included cohort studies.

Study	Selection	Comparability	Outcome	NOS stars
Abbey and Farrow (1998a,b)	***		*	4
Baines et al. (2002a,b)	****	*	***	8
Baines et al. (2004)	****	**	**	8
Buchanan (1975)	***		**	5
Carrique et al. (2021)	****	**	***	9
De Geest et al. (2006)	****	**	***	9
Ghetti (2011)	***		**	5
Gross et al. (2009)	***	*	***	7
Hogan and Silverman (2015)	****	*	**	7
Kober et al. (1990)	**		*	3
Lisson et al. (2005)	***		**	5
Stonnington et al. (2016)	***	*	**	6
Wagner-Skacel et al. (2023)	****	*	***	8

**Table 3:** Quality assessment of included cross sectional studies.

Study	Selection	Comparability	Outcome	NOS stars
Epstein et al. (2019a,b)	***	*	**	
Freyberger et al. (1979)	****	*	**	7
Heilmann et al. (2011)	****	*	***	8
Hodges et al. (1995)	***		**	5
Madson and Silverman (2010)	**		**	4

## Results

### Literature search results

A comprehensive database search was conducted in PubMed, Scopus, and Web of Science, yielding a total of 89 records. After removing 24 duplicates, 65 unique articles remained. Following title and abstract screening, eight studies were excluded (six lacking abstracts and two involving pediatric or partially pediatric populations).

Full-text assessment was performed for 57 studies. Of these, 33 were excluded: three were published in non-eligible languages (German or Spanish), 19 full texts could not be retrieved despite repeated attempts through institutional library services and author contact, and eleven lacked validated or adequately described psychological interventions.

Ultimately, 24 studies met the inclusion criteria and were incorporated into the qualitative synthesis. Table 1 summarizes the included studies, specifying design characteristics, intervention types, duration, and main outcomes.

### Included studies

The reviewed literature provides a heterogeneous yet convergent body of evidence regarding psychological interventions in the context of organ transplantation. Despite methodological variability and generally moderate NOS scores across studies, most investigations reported improvements in psychological well-being, treatment adherence, and quality of life. However, several studies suffered from limited comparability due to the absence of control groups, small samples, or heterogeneous outcome measures, which influences the strength of the conclusions drawn.

### Cognitive-behavioral therapy (CBT) and related approaches

Cognitive-Behavioral Therapy (CBT) emerged as the most empirically supported approach across the included studies, although the overall quality of evidence varied. Higher-quality cohort studies, such as those by Carrique et al. (2021) and De Geest et al. (2006), which obtained eight or nine stars on the NOS, provided more reliable findings, with clear intervention protocols, adequate follow-up, and satisfactory outcome assessment. In Carrique et al. (2021) CBT significantly reduced depression scores at 6 months (*p* = 0.003) and anxiety (*p* = 0.011), and improved quality of life (*p* = 0.027). Similarly, De Geest et al. reported significant improvements in emotional well-being (*p* = 0.005), social functioning (p = 0.01), and a reduction in illness intrusiveness (*p* = 0.007).

CBT and CBT-derived models—including Motivational Enhancement Therapy (MET) and Relapse Prevention Therapy (RPT)—were effective in reducing craving, preventing relapse, and improving adherence among liver transplant candidates and recipients with alcohol use disorder (Heather and Stockwell, 2004; Weinrieb and Lucey, 2007). Remote delivery of CBT via telehealth also demonstrated reductions in depression and anxiety among heart transplant recipients (Epstein et al., 2019a,b), though this evidence stemmed from lower-quality cross-sectional studies. Nonetheless, Epstein et al. (2019a,b) showed significant reductions in BDI scores post-intervention (*p* = 0.001), with effects maintained at 12-month follow-up.

Although some of the evidence is limited by selection and reporting bias, the consistent statistical significance across designs supports the clinical utility of CBT interventions in transplant populations.

### Group psychotherapy

Group-based interventions consistently fostered peer connection, emotional sharing, and support, facilitating reductions in anxiety and depressive symptoms. Nonetheless, the overall quality of evidence in this area was limited: most studies showed moderate-to-high risk of bias, with NOS scores ranging from 4 to 5 stars. These limitations arose primarily from small samples, absence of control groups, lack of standardized outcome measures, and reliance on subjective reports.

Early contributions such as Buchanan (1975) and Abbey and Farrow (1998a,b) documented improved emotional support and reduced isolation, but these studies did not report statistical tests and relied on qualitative feedback. More structured evidence emerged in Baines et al. (2004), where depressive symptoms improved significantly in the group-therapy arm (BDI 30.2 → 26.0; *p* = 0.01). Even though individual therapy demonstrated stronger effects, the presence of significant changes in the group condition supports the usefulness of group formats despite their methodological fragility.

Overall, despite the moderate risk of bias characterizing most studies in this category, the convergence of findings suggests that group interventions meaningfully enhance emotional expression, perceived support, and communication among transplant recipients.

### Psychoeducational interventions

Psychoeducational programs demonstrated consistent benefits in supporting adjustment, coping, self-efficacy, and adherence. Evidence in this domain was generally stronger, with several studies achieving high methodological quality, including NOS scores of 8–9 stars (e.g., Chisholm-Burns et al., De Geest et al., 2006). These studies offered more standardized measures, clearer intervention descriptions, and objective adherence assessments.

De Geest et al. (2006) reported improved adherence in the intervention group, with a significant effect (*p* = 0.06, borderline significance given the small sample). Chisholm-Burns et al. (2013), although not reporting *p*-values, documented clear objective improvements in medication adherence. In contrast, bibliotherapy (Hodges et al., 1995), supported by cross-sectional evidence with moderate risk of bias, nonetheless showed significant reductions in emotional expression (*p* < 0.01) and social withdrawal (*p* < 0.0001).

Taken together, psychoeducational interventions present the strongest methodological base among non-CBT approaches, although heterogeneity in formats and follow-up duration indicates that long-term impacts remain underexplored.

### Expressive and experiential therapies

Expressive therapies—including music therapy—showed significant reductions in anxiety, pain, and stress. However, evidence in this domain was derived largely from small-sample cohort or cross-sectional studies with moderate risk of bias (NOS 5–7 stars), reflecting limited comparability, absence of blinding, and reliance on self-reported outcomes.

Ghetti (2011) reported significant reductions in anxiety (*p* = 0.005) and anger expression (*p* = 0.047), along with improved physical role functioning (*p* = 0.036) after 12 weeks of music therapy. Similarly, Madson and Silverman (2010) documented significant improvements in relaxation and anxiety (*p* < 0.001), pain (p < 0.01), nausea (*p* < 0.05), and positive affect (p < 0.001) immediately after a single session.

Despite the methodological constraints limiting generalizability, the repeated pattern of significant emotional and somatic improvements suggests that expressive therapies offer valuable adjunctive benefits in transplant care.

### Mindfulness-based interventions

Mindfulness-based interventions, including MBSR and MBRT, consistently produced significant reductions in anxiety, depression, perceived stress, and insomnia. The methodological quality of studies in this category was moderate (NOS 6–7 stars), with limitations including small sample sizes, non-randomized designs, and short follow-up durations, increasing the risk of selection and attrition bias.

Gross et al. (2009) found significant reductions in depression (p < 0.001), anxiety (p < 0.001), and insomnia (*p* = 0.001), maintained at 6 and 12 months. Stonnington et al. (2016) similarly reported significant reductions in perceived stress (*p* = 0.0011), depression (*p* = 0.0025), anxiety (*p* = 0.0002), negative affect (*p* = 0.0027), and improvements in positive affect (*p* = 0.0056) and mental quality of life (*p* = 0.0247).

Although methodological constraints temper the strength of conclusions, the consistency of statistically significant improvements across domains strengthens confidence in the efficacy of mindfulness-based approaches.

### Interpersonal and psychodynamic approaches

Interpersonal and psychodynamic interventions were supported by case studies and small observational research with low-to-moderate methodological quality (NOS 3–5 stars). These designs inherently carry substantial risk of bias—particularly selection, reporting, and ascertainment bias—and typically lacked statistical testing.

No *p*-values were reported in these studies. Nonetheless, qualitative findings highlighted clinically important themes including identity reconstruction, guilt, and emotional integration post-transplantation. While generalizability is limited, these observations capture aspects of the transplant experience often overlooked by more structured approaches.

### Multidisciplinary and integrated interventions

Integrated psychological care models demonstrated improvements in adherence, psychiatric symptoms, and overall patient–provider collaboration. Evidence quality varied widely: some older studies (e.g., Kober et al., 1990) received low NOS scores (3 stars), indicating substantial methodological limitations. In contrast, more recent research, such as Wagner-Skacel et al. (2023), achieved high methodological quality (8 stars), benefitting from objective adherence monitoring and structured follow-up.

Wagner-Skacel et al. reported significantly lower non-adherence in the intervention group (*p* = 0.019), fewer hospital readmissions (*p* = 0.049), and reduced tacrolimus variability (*p* = 0.028). Despite heterogeneity in study designs, multidisciplinary interventions overall appear well-positioned to support long-term psychosocial adjustment.

## Discussion

### Summary of main findings

The analysis of the twenty included studies highlights the central role of psychological and psychosocial interventions throughout the transplant trajectory, addressing anxiety, depression, adherence, and post-surgical adjustment. Despite the diversity of approaches—ranging from cognitive-behavioral and psychoeducational to mindfulness-based, expressive, and psychodynamic modalities—a consistent pattern emerges regarding the benefits of structured, evidence-based psychological care. However, the overall methodological quality of the available evidence remains heterogeneous, with NOS scores indicating moderate risk of bias in several domains and more robust evidence concentrated primarily within CBT and structured psychoeducational interventions.

Cognitive Behavioral Therapy (CBT) and CBT-derived interventions consistently demonstrated the strongest empirical support. Across multiple studies (Carrique et al., 2021; Epstein et al., 2019a,b; Parker Gregg, 2021), CBT was associated with significant reductions in depressive and anxiety symptoms and improvements in adherence, findings that were supported by clearer intervention protocols, objective outcome assessments, and higher NOS scores. These characteristics contributed to lower risk of bias relative to other intervention types. The effectiveness of CBT appears to stem from its capacity to address maladaptive cognitions and behaviors commonly observed in transplant recipients—such as health-related anxiety, fear of rejection, and guilt toward the donor—while reinforcing adaptive coping strategies and self-efficacy (Heather and Stockwell, 2004). These mechanisms directly influence both psychological well-being and clinical outcomes, promoting treatment adherence and, indirectly, graft stability. Moreover, the flexibility of CBT enables its integration within multidisciplinary care teams and its delivery through both in-person and telehealth modalities.

These results align with the conclusions of the recent meta-analysis by Sambucini et al. (2022), which identified CBT as the most empirically supported framework for transplant populations but emphasized the need for studies with greater methodological rigor. The present findings reinforce this evidence, showing that CBT’s structured, goal-oriented framework is particularly suited to address the cognitive and behavioral patterns that compromise adjustment and adherence after transplantation.

Group-based and psychoeducational interventions also showed meaningful benefits, fostering peer identification, emotional sharing, and perceived support—key elements for patients dealing with post-transplant identity reorganization and ambivalence toward the donor. However, unlike CBT, most group-based studies were characterized by small sample sizes, reliance on subjective measures, and lower NOS scores, indicating higher risk of bias. Psychoeducational interventions, particularly structured adherence programs such as SMART (De Geest et al., 2006) and Behavioral Adherence Contracts (Chisholm-Burns et al., 2013), demonstrated more solid methodological foundations, including objective adherence monitoring and clearer follow-up procedures.

Mindfulness-based and expressive therapies contributed additional value by reducing anxiety, improving emotional processing, and enhancing quality of life. These interventions showed several statistically significant improvements, yet they were generally supported by studies of moderate methodological quality, often constrained by small samples, limited randomization, and short follow-up periods. Their patient-centered nature makes them promising adjunctive interventions, although further rigorous studies are needed to clarify their long-term impact.

Finally, psychodynamic and interpersonal approaches, though represented mainly by small observational studies and case reports, provided meaningful insight into deeper psychological dimensions such as guilt, identity reconstruction, and psychological “organ rejection.” While the high risk of bias and lack of statistical testing limit generalizability, these findings underscore the complexity of post-transplant psychological adaptation and the relevance of approaches that address identity and meaning-making processes.

Taken together, these findings confirm that CBT currently represents the most empirically validated and clinically adaptable intervention for transplant recipients, due to its robust theoretical foundation, structured methodology, and demonstrated improvements across emotional and behavioral domains in studies with higher methodological quality. However, consistent with Sambucini et al. (2022), the literature remains limited by small samples, brief follow-ups, and methodological heterogeneity, restricting the generalizability of results. Future research should therefore prioritize multicenter, longitudinal studies comparing psychological approaches using standardized outcome measures and integrating both psychological and clinical endpoints such as adherence, rejection episodes, and quality of life.

From a clinical perspective, incorporating CBT-informed modules within routine transplant care could represent a feasible and evidence-based strategy to enhance long-term adjustment, adherence, and biopsychosocial well-being. Additional efforts should also focus on improving methodological quality across all intervention types to strengthen the empirical foundation supporting psychological care in transplant populations.

## Limitations and critical issues of the study

Although the reviewed studies highlight the relevance of psychological interventions for transplant patients, several methodological and clinical limitations emerged, which may affect the reliability and generalizability of the findings.

### Sample size and study design

A recurrent issue concerns the small sample sizes in most studies, which limit statistical power and the possibility of drawing robust conclusions. Many investigations involved fewer than fifty participants, sometimes as little as a single clinical case (e.g., Freedman, 1983; Miller, 2002). In addition, several studies lacked a control group or randomization, making it difficult to establish whether the observed improvements were specifically attributable to the intervention or to external factors such as natural recovery or medical stabilization.

### Duration of follow-up

Another critical limitation is the short follow-up period. While several interventions, such as CBT or mindfulness programs, demonstrated positive outcomes in reducing anxiety, depression, and distress, these effects were often measured only at the end of treatment or within a few months thereafter (Gross et al., 2010; Epstein et al., 2019a,b). Very few studies assessed the long-term sustainability of psychological improvements, which is particularly important in transplantation, where adherence and emotional adjustment are lifelong challenges.

### Heterogeneity of populations and interventions

The included studies encompassed heterogeneous populations, involving recipients of different organs (kidney, liver, heart, lung) and varying psychosocial backgrounds. Similarly, the interventions ranged widely—from psychoeducation and bibliotherapy to psychoanalysis, music therapy, and tele-CBT. While this variety reflects the complexity of transplant care, it also hinders direct comparisons between interventions and the identification of specific treatment indications for different patient subgroups.

### Database selection

A methodological limitation of this review lies in the database selection. Although PubMed, Scopus, and Web of Science provide broad coverage, the exclusion of other specialized databases could have potentially resulted in the failure to include a small number of highly specific studies, particularly those less indexed in multidisciplinary sources.

### Measurement tools and outcome evaluation

Although some studies employed standardized psychometric instruments such as the Beck Depression Inventory (BDI) or the Hospital Anxiety and Depression Scale (HADS), others relied on self-report questionnaires or non-validated tools. This heterogeneity in outcome measures complicates cross-study comparisons and raises concerns about the consistency of reported effects. Moreover, several studies focused exclusively on psychological outcomes without integrating medical data such as graft survival, immunosuppressant levels, or hospitalizations—factors that are essential in evaluating the clinical impact of psychological interventions.

### External and contextual factors

Another limitation lies in the insufficient consideration of external variables that may influence treatment outcomes. Elements such as family support, socioeconomic status, and pre-existing psychiatric conditions were rarely analyzed systematically. These factors, however, are known to play a crucial role in adherence and emotional adaptation post-transplant, and their omission weakens the ecological validity of the findings.

For instance, physical frailty has been associated with poorer post-transplant quality of life (McAdams-DeMarco et al., 2018), and individual coping strategies can significantly affect psychological outcomes (Suzuki et al., 2019); yet such variables were largely unexamined in the reviewed studies. Moreover, unforeseen external events like the COVID-19 pandemic have imposed additional psychosocial burdens on transplant patients, with recent studies documenting elevated distress and anxiety in this population during the pandemic (Baey et al., 2022; De Pasquale et al., 2024; Ford et al., 2024; Gundogmus et al., 2023; Zhang et al., 2025).

## Conclusion

Organ transplantation is not merely a life-saving medical procedure but a profoundly transformative experience that reshapes the patient’s emotional, psychological, and social landscape. The evidence synthesized in this review demonstrates that psychotherapeutic interventions—particularly those grounded in cognitive-behavioral principles—play a pivotal role in promoting adherence, enhancing quality of life, and supporting the complex process of post-transplant adjustment.

Among the available approaches, Cognitive Behavioral Therapy (CBT) emerges as the most empirically supported and clinically adaptable intervention. Its structured, goal-oriented techniques effectively address the cognitive distortions and maladaptive behaviors that often undermine adherence and psychological well-being in transplant recipients, thereby improving both mental health and medical outcomes.

Moving forward, future research should prioritize multicenter and longitudinal studies to validate these findings, refine intervention protocols, and determine which psychological approaches best suit specific clinical profiles. Integrating evidence-based psychological care—especially CBT-informed interventions—into standard transplant pathways represents not only a scientific and clinical priority but also a necessary step toward achieving truly holistic and patient-centered care. Finally, it is hoped that future studies on psychological interventions in organ transplantation will adopt greater homogeneity in methodology, therapeutic approaches, and primary outcomes, thus allowing subsequent reports to use meta-analyses for a more robust pooled estimate of effectiveness.

## Data Availability

The raw data supporting the conclusions of this article will be made available by the authors, without undue reservation.

## Footnotes

1
www.ohri.ca/programs/clinical_epidemiology/oxford.asp
